# Association between the hyperuricemia and nonalcoholic fatty liver disease risk in a Chinese population: A retrospective cohort study

**DOI:** 10.1371/journal.pone.0177249

**Published:** 2017-05-16

**Authors:** Chao Yang, Shujuan Yang, Weiwei Xu, Junhui Zhang, Wenguang Fu, Chunhong Feng

**Affiliations:** 1 Department of Epidemiology and Health statistics, School of Public Health, Southwest medical University, Luzhou, China; 2 Department of Health Related Social and Behavioral Science, West China School of Public Health, Sichuan University, Chengdu, China; 3 Health Management Department, the First Affiliated Hospital, College of Medicine, Southwest Medical University, Luzhou, China; 4 Department of Hepatobiliary Surgery the First Affiliated Hospital, College of Medicine, Southwest Medical University, Luzhou, China; Universita degli Studi di Verona, ITALY

## Abstract

Nonalcoholic fatty liver disease (NAFLD) is a common chronic disease associated with high levels of serum uric acid (SUA). However, whether this relationship applies in obese subjects has been unclear, and no cohort study has previously been conducted in non-obese subjects. We therefore performed a retrospective cohort study among employees of seven companies in China to investigate whether hyperuricemia was independently associated with NAFLD in obese and non-obese subjects, respectively. A total of 2383 initially NAFLD-free subjects were followed up for four years, and 15.2% (363/2383) developed NAFLD. Hyperuricemia subjects had a higher cumulative incidence than did those with normouricemia (29.0% vs. 12.9%, *P*<0.001). Cox proportional hazard regression analyses showed that baseline hyperuricemia was significantly associated with risk of developing NAFLD in non-obese subjects. This relationship was significantly independent of baseline age, gender, metabolic syndrome components, and other clinical variables (RR = 1.389, 95%CI: 1.051–2.099). However, this association did not exist in obese subjects (RR = 1.010, 95%CI: 0.649–1.571). The independent effect of hyperuricemia on NAFLD was stronger in females (RR = 2.138, 95%CI: 1.050–4.355) than in males (RR = 1.435, 95%CI: 1.021–2.018). In conclusion, further studies are needed to explore the different mechanisms between obese and non-obese subjects, and the reason hyperuricemia raises NAFLD risk in females more than in males.

## Introduction

Nonalcoholic fatty liver disease (NAFLD) is caused by accumulation of fat in the cytoplasm of liver cells, and not by excessive alcohol consumption or other known liver pathologies [1]. It includes simple steatosis and nonalcoholic steatohepatitis (NASH), which can progress to cirrhosis and its associated complications [2, 3]. NAFLD is the most prevalent chronic liver disease in Western countries, and is an increasingly serious health problem in Asia, where subjects tend to be less obese. It affects 24–42% of the general population in Western countries and 5–42% in Asian countries [4–8]. Patients with NAFLD have a markedly higher risk of death compared with the general population [9, 10]. In addition, NAFLD can increase the risk of cardiovascular disease [11, 12].

Serum uric acid(SUA) is the end product of purine metabolism in the human body and originates from hypoxanthine after double enzyme catalysis by xanthine oxidase in the liver [13]. It is a metabolic product of endogenous(nucleoproteins originating from cellular metabolism)and an exogenous (dietary) precursor protein delivered to the liver, and is excreted by the kidneys. Therefore, any impairment in this balance can lead to high SUA levels [14, 15]. The relationship between SUA and NAFLD was first described in a small Italian study in 2002 [16]. Evidence for the relationship was then strengthened by a number of cross-sectional studies [17–28] and several cohort studies [29–31] in which SUA was reported to be an independent risk factor for NAFLD. Although these studies involved a variety of populations, the mechanism linking SUA and NAFLD had not been fully elucidated. Moreover, to the best of our knowledge, no cohort study specifically examined the relationship between SUA levels and NAFLD in obese subjects. Thus, further studies are necessary to explore this potential relationship, which may be helpful in elucidating the mechanism of NAFLD.

Four cross-sectional studies [25, 32–34]on the relationship between SUA and NAFLD in non-obese subjects were conducted between 2006 and 2016.Non-obese was defined as BMI<25 kg/m^2^ because all of the studies(three of Chinese and one of Korean subjects) took place in Asian populations, which tend to have relatively low rates of obesity. Surprisingly, these studies all suggested that high SUA levels are an independent risk factor for NAFLD in non-obese subjects. However, the existence of such a relationship needs to be confirmed in a cohort study.

Therefore, in this retrospective cohort study, we aimed to clarify whether hyperuricemia was independently associated with the development of NAFLD in obese and non-obese subjects, and whether obesity influences the relationship between NAFLD and SUA.

## Materials and method

### Ethics statement

Verbal informed consent was obtained from each subject before participation in the physical examination. The study was approved by the Ethics Committee of the First Affiliated Hospital, Clinical Medicine College, Southwest medical University.

### Study design and subjects

**Fig 1:**
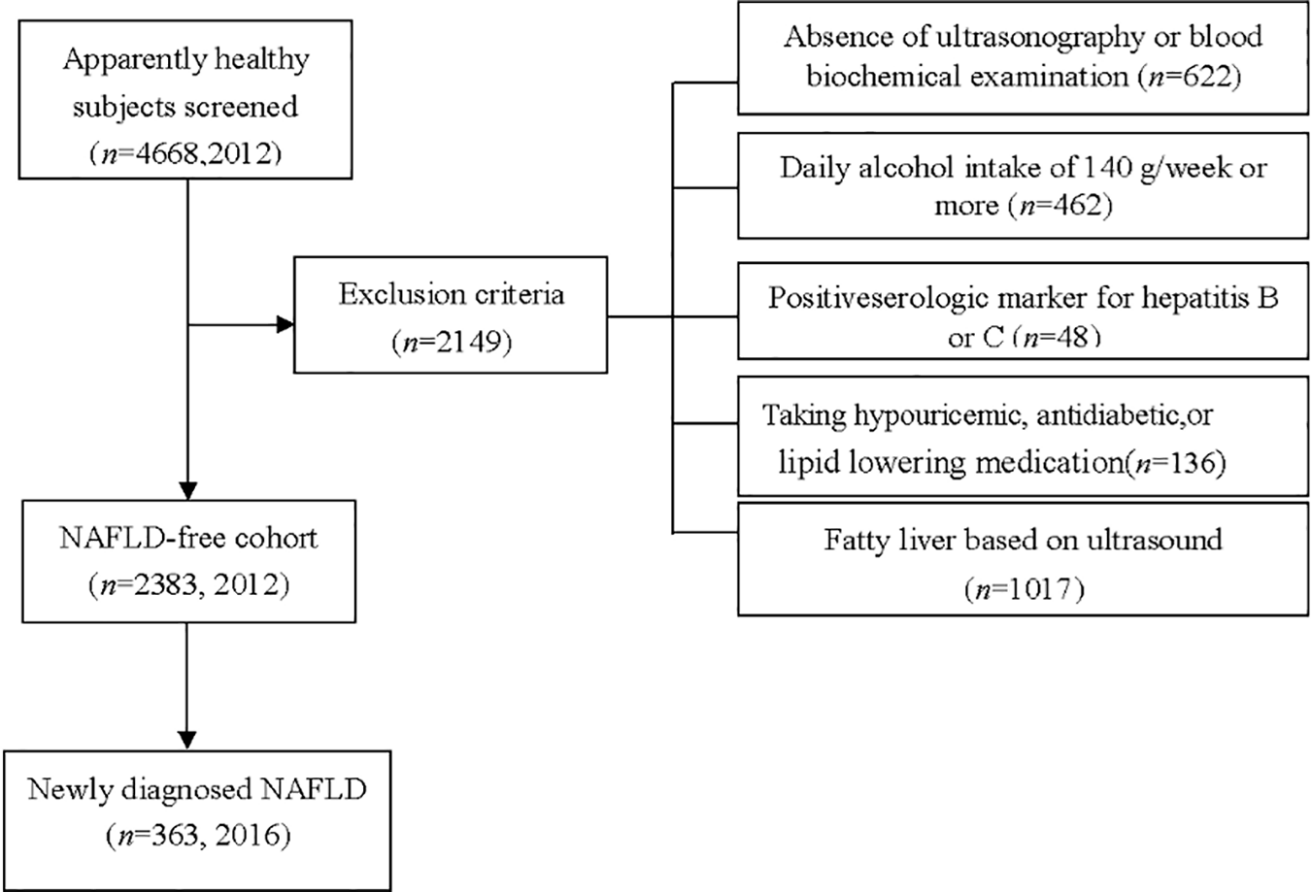
Flow diagram of subjects’ selection. A total of 4668 employees underwent health screening in 2012, of which 2149 subjects were excluded due to following reasons: absence of ultrasonography or blood biochemical examination; with heavy drinking (daily alcohol intake of 140 g/week or more); with a positive serologic marker for hepatitis B or C; diagnosed with fatty liver based on ultrasonography; taking hypouricemic, antihypertensive, antidiabetic and lipid lowering medication. Consequently, 2383 subjects were observed for incident NAFLD after 4 years.

To identify whether SUA was independently associated with the development of NAFLD in obese and non-obese subjects, and compare the role of SUA in the two types of subjects, a retrospective cohort study was conducted in 2016. A total of 4668 employees from seven companies were screened for our study based on the results of health examinations conducted in 2012 and 2016, at the first Affiliated Hospital of Southwest Medical University, Luzhou, China. All subjects performed overall baseline assessment in 2012 and were observed for NAFLD development after 4 years in 2016. Of these, 2149 of the subjects were excluded for the following reasons: 622 lacked ultrasonography or blood biochemical examinations; 462 were heavy drinkers (defined as intake of 140 g/week or more); 48 tested positive for serologic markers of hepatitis B or C; 1017 were diagnosed with fatty liver based on ultrasonography in 2012; and 136 were taking hypouricemic, antihypertensive, antidiabetic, or lipid lowering medications (Fig 1).

Ultimately, a total of 2383 initially NAFLD-free subjects (1189 males and 1194 females) comprised this cohort study and were retrospectively observed for the development of NAFLD for four years.

### Baseline examinations

Baseline examinations included a health habit inventory, anthropometric measurements, hepatic ultrasonic examination and biochemical measurements.

Systolic blood pressure (SBP) and diastolic blood pressure (DBP) were measured using an automated sphygmomanometer with the subject in a sitting position. Standing height and body weight were measured without shoes or outer clothing. Body mass index (BMI, kg/m^2^) was calculated as weight in kilograms divided by height in meters squared.

The examinations were administered between 8:00–11:00 a.m. Participants were instructed to fast for at least 12 hours prior to the examination and to refrain from exercise the day before. The biochemical parameters measured included levels of total cholesterol (TC), triglycerides (TG), high-density lipoprotein cholesterol (HDL-c), low-density lipoprotein cholesterol (LDL-c), fasting plasma glucose (FPG), creatinine (Cr), blood urea nitrogen (BUN), serum uric acid (SUA), aspartate aminotransferase(AST) and alanine aminotransferase (ALT). All biochemical parameters were measured by an automatic biochemical analyzer (SIEMENS ADVIA2400).

### Assessment of outcomes and definitions

The definition of obese in this study was based on the Asian populations in previous studies (BMI ≥25) [25, 32–34]. Hyperuricemia (**hyper**) was defined as an SUA level of >420 *μ*mol/L in males and >360 *μ*mol/L in females, and subjects with SUAs below these cutoffs were classed as normouricemia(**normo**)[35].

Diagnosis of fatty liver was based on the results of abdominal ultrasonography using a GE Logig 9 sonography machine with a 10.0-MHz probe. Ultrasound examinations were carried out by two senior imaging specialists. Hepatic steatosis was diagnosed by characteristic echo patterns according to conventional criteria, such as the evidence of diffuse hyperechogenicity of the liver relative to the kidneys, ultrasound beam attenuation and poor visualization of intrahepatic structures [36]. NAFLD was diagnosed by abdominal ultrasound following exclusion of alcohol consumption, viralor autoimmune liver disease[37].

### Statistical analyses

Continuous variables are expressed as means±SD or medians and inter-quartile ranges, and were compared using the Student t-test or the Mann-Whitney U test, depending on the normality of the data. Categorical variables were compared using the chi-square test.

The Cox proportional hazards regression model was used to estimate hazard ratios for incident NAFLD for hyperuricemia. The analysis was carried out in two steps: First, the effect of hyperuricemia was estimated in a univariate model; second, baseline age, gender, TC, TG, HDL-c, LDL-c, FPG, Cr, BUN, SUA, AST, ALT, SBP, DBP and BMI were adjusted in a multivariate model. The Cox proportional hazards regression analysis was divided into two steps. First, the analysis was carried out for the total population. Second, the analysis was performed on the two subgroups (non-obese and obese), and a stratified analysis was conducted by gender in each subgroup.

All statistical analyses were performed using the SPSS software package version 17.0 for Windows. P <0.05 (2-tailed) was considered to be statistically significant.

## Results

### Baseline characteristics

At baseline, the mean age (±SD, range) of the 2383 participants was 40.8 years (±12.2, 19–93). The hyper group consisted of 341 subjects who had high SUA levels, while the other 2042 subjects with normal SUA levels comprised the normo group. The baseline characteristics of subjects in the two groups are shown in Table 1. Average age, TC, TG, LDL-C, Cr, ALT, AST, SBP, DBP, and BMI were all higher in the hyper group than in the normo group (*P*<0.001). In contrast, the average HDL-c level was lower in the hyper group (*P*<0.001). The difference in the gender ratio was significant between the two groups; the hyperuricemia group had a higher constituent ratio of males than did the normouricemia group (*P*<0.001). No significant difference was observed in FPG between the two groups.

**Table 1:** Baseline characteristics of subjects with and without hyperuricemia.

Variable	All subjects *n* = 2383	Normo *n* = 2042	Hyper *n* = 341	*t*	*P*
Gender, male/female	1189/1194	911/1131	278/63	159.250 ^a^	<0.001
Age (years)	40.80±12.18	40.41±11.74	43.14±14.34	3.334	<0.001
TC (mmol/L)	4.67±0.85	4.65±0.84	4.84±0.93	3.839	<0.001
FPG (mmol/L)	5.00±0.89	5.00±0.89	5.04±0.92	0.831	0.406
TG (mmol/L)	1.15 (0.85–1.58)	1.11 (0.83–1.49)	1.48 (1.12–2.15)	11.126^b^	<0.001
HDL-C (mmol/L)	1.50±±0.34	1.52±0.34	1.36±0.32	7.991	<0.001
LDL-C (mmol/L)	2.44±0.67	2.42±0.66	2.57±0.71	3.767	<0.001
BUN (mmol/L)	5.00±1.29	4.94±1.22	5.38±1.62	4.833	<0.001
Cr (*μ*mol/L)	66.90 (56.40–79.10)	64.50 (55.10–76.70)	78.90 (70.30–88.70)	14.458^b^	<0.001
SUA (mg/dl)	5.38±1.37	4.99±1.00	7.71±0.90	50.631	<0.001
ALT (*μ*mol/L)	18.70 (14.30–26.60)	18.00 (14.00–25.30)	24.60 (17.60–33.75)	9.531^b^	<0.001
AST (*μ*mol/L)	23.10 (19.90–27.40)	22.70 (19.60–26.83)	26.00 (22.70–30.95)	9.091^b^	<0.001
SBP (mmHg)	115.59±17.49	113.99±16.91	125.21±17.83	10.820	<0.001
DBP (mmHg)	77.62±10.97	76.74±10.67	82.91±11.24	9.794	<0.001
BMI (kg/m^2^)	21.79±2.58	21.58±2.55	23.00±2.44	9.525	<0.001

Data are expressed as the means±SD or medians (25th to 75th percentiles). The subjects were grouped according to serum uric acid: normo and hyper. Two groups were compared with student-t-test, chi-square test and Mann-Whitney U test. TC, total cholesterol; FPG, fasting plasma glucose; TG, triglyceride; HDL-c, high-density lipoprotein cholesterol; LDL-c, low-density lipoprotein cholesterol; BUN, blood urea nitrogen; Cr, creatinine; AST, aspartate aminotransferase; ALT, alanine aminotransferase; SBP, systolic blood pressure; DBP, diastolic blood pressure; BMI, body mass index.

^a^ value

^b^ Z value

### Hyperuricemia increases the risk of NAFLD

Over the four-year study period, 363 subjects (256 male and 107 female) developed NAFLD, a 21.5% and 9.0% cumulative incidence in men and women, respectively. The overall four-year cumulative incidence of NAFLD was15.2%, and those with hyperuricemia had a significant higher incidence of NAFLD than those with normouricemia (29.0% vs. 12.9%, *P*<0.001). Hyperuricemia was an independent risk factor for NAFLD both in univariate (RR = 2.246, 1.782–2.829) and multivariate models (RR = 1.416, 1.103–1.819).

Among males, the hyper group had a significantly higher incidence of NAFLD than did the normo group (30.9% vs. 18.7%, *P*<0.001). The relationship between hyperuricemia and NAFLD was significant both in univariate (RR = 1.658, 1.279–2.149) and multivariate models (RR = 1.389, 1.057–1.824). In female subjects, the hyper group also had a significantly higher incidence of NAFLD than the normo group (20.6% vs. 8.3%). However, the significant relationship only existed in the univariate model (RR = 2.483, 1.390–4.434) (Table 2).

**Table 2:** Univariate and multivariate relationships between baseline hyperuricemia and incidence of NAFLD (*n* = 2383).

Population	Group	NAFLD	*n*	%	*RR*	95% CI	*RR*_Adj_	95% CI_Adj_
All (*n* = 2383)	Normo	264	2042	12.9	1		1	
	Hyper	99	341	29.0	2.246	1.782–2.829	1.416	1.103–1.819
Male (*n* = 1189)	Normo	170	911	18.7	1		1	
	Hyper	86	278	30.9	1.658	1.279–2.149	1.389	1.057–1.824
Female (*n* = 1194)	Normo	94	1131	8.3	1		1	
	Hyper	13	63	20.6	2.483	1.390–4.434	1.667	0.890–3.123

*RR*, relative risk of nonalcoholic fatty liver disease; CI, confidence interval; *RR*_adj_, RR in multivariable models; CI_adj_, CI in multivariable models.

### Hyperuricemia increases the risk of NAFLD in non-obese subjects

Among non-obese subjects, those in the hyper group had a significantly higher incidence of NAFLD than those with normouricemia (25.2% vs. 10.2%, *P*<0.001). Hyperuricemia was an independent risk factor for NAFLD both in univariate (RR = 2.463, 1.867–3.249) and multivariate models (RR = 1.542, 1.134–2.099). This relationship was still significant when the non-obese subjects were divided into men (RR = 1.435, 1.021–2.018) and women (RR = 2.138, 1.050–4.355). The results suggested that the effect of hyperuricemia on NAFLD is larger in females than in males (Table 3).

**Table 3:** Univariate and multivariate relationship between baseline hyperuricemia and incidence of NAFLD in non-obese subjects.

Population	Group	NAFLD	*n*	%	*RR*	95% CI	*RR*_Adj_	95% CI_Adj_
All (*n* = 2128)	Normo	190	1858	10.2	1		1	
	Hyper	68	270	25.2	2.463	1.867–3.249	1.542	1.134–2.099
Male (*n* = 999)	Normo	114	782	14.6	1		1	
	Hyper	58	217	26.7	1.833	1.337–2.515	1.435	1.021–2.018
Female (*n* = 1066)	Normo	76	1076	7.1	1		1	
	Hyper	10	53	18.9	2.671	1.382–5.165	2.138	1.050–4.355

*RR*, relative risk of nonalcoholic fatty liver disease; CI, confidence interval; *RR*_adj_, RR in multivariable models; CI_adj_, CI in multivariable models.

### Hyperuricemia was not associated with NAFLD in obese subjects

The relationship between hyperuricemia and NAFLD was not significant in obese subjects in either the univariate or multivariate models. This relationship was still not significant when the obese subjects were analyzed separately by gender (Table 4).

**Table 4:** Univariate and multivariate relationship between baseline hyperuricemia and incidence of NAFLD in obese subjects.

Population	Group	NAFLD	*n*	%	*RR*	95% CI	*RR*_Adj_	95% CI_Adj_
All (*n* = 255)	Normo	74	184	40.2	1		1	
	Hyper	31	71	43.7	1.086	0.714–1.651	1.010	0.649–1.571
Male (*n* = 332)	Normo	56	129	43.4	1			
	Hyper	28	61	45.9	1.057	0.672–1.664	1.095	0.678–1.768
Female (*n* = 128)	Normo	18	55	32.7	1		1	
	Hyper	3	10	30.0	0.917	0.270–3.312	0.683	0.116–4.028

*RR*, relative risk of nonalcoholic fatty liver disease; CI, confidence interval; *RR*_adj_, RR in multivariable models; CI_adj_, CI in multivariable models.

## Discussion

Since the pioneering studies inaugurating the line of research linking NAFLD with increased SUA levels, an increasing number of studies, including several cross-sectional studies [17–27], three cohort studies [29–31] and two meta-analytic reviews [38, 39], have confirmed that high SUA levels was an independent risk factor of NAFLD. In addition, other studies enriched the relationship between SUA levels and NAFLD. Mosca A et al. [40] found that both SUA concentrations and dietary fructose consumption were independently associated with NASH in children and adolescents, and SUA concentrations were associated with dietary fructose consumption. Another recent meta-analysis showed that hyperuricemia was not associated with severity of liver fibrosis in patients with nonalcoholic fatty liver disease[41]. Lombardi et al.[42]summarized the accumulated evidence and suggested that SUA may contribute to NAFLD pathogenesis mainly through its interaction with insulin resistance[43], induced radical oxygen species(ROS)[44] and activation of the NLRP3 inflammasome [45]. Although specific pathogenesis of NAFLD induced by high SUA levels is still unclear, the conventional paradigm of NAFLD representing the "hepatic manifestation of the metabolic syndrome" is becoming outdated. Lonardo et al. summarized the results of numerous studies and supported the novel paradigm of NAFLD as a strong determinant for the development of the metabolic syndrome, which has potentially relevant clinical implications for diagnosing, preventing and treating metabolic syndrome[46]. Italian Association for the Study of the Liver (AISF) also concluded that the results of ongoing studies are eagerly expected to lead to introduce into the clinical arena new diagnostic and prognostic biomarkers, prevention and surveillance strategies as well as to new drugs for a tailored approach to the management of NAFLD in the individual patient[47]. In this four-year retrospective cohort study, we observed that baseline hyperuricemia was positively and significantly associated with NAFLD risk in initially NAFLD-free subjects. This relationship was significant independent of baseline age, gender, metabolic syndrome components and other clinical variables, but it was found only in non-obese subjects, not in obese subjects. The independent effect of hyperuricemia on NAFLD was higher in women than in men. Our results confirm the relationship between high SUA levels and development of NAFLD, and add detail to it.

In 2007, a study of 268 obese children in Italy found that uric acid was an independent predictor of NAFLD [48]. Recently, another study of 1365 obese Chinese adults confirmed this, demonstrating that SUA is independently and linearly associated with risk of NAFLD[24]. Interestingly, a Brazilian study reached the opposite conclusion: that high levels of uric acid were not associated with NAFLD in overweight or obese children and adolescents [49].The relationship between SUA and NAFLD in obese people has therefore been unclear. However, hyperuricemia was not associated with NAFLD in obese subjects in this study. It was not consistent with the earlier Chinese study in which elevated SUA was independently associated with increased risk of NAFLD in obese adults, with an adjusted OR of 1.528–2.031 [24]. The reasons for the different findings may include the larger sample size (2383 vs. 1365), younger subjects(40.8±12.2 vs. 53.4±7.3) and lower proportion of women(50.1% vs. 69.2%). The largest distinction between the studies was that subjects with NAFLD were excluded at baseline in our retrospective cohort study, but not in the previous study. Thus, high SUA levels were observed before NAFLD in initially NAFLD-free subjects.

It was well known that obesity is a direct risk factor for NAFLD. However, lean individuals with NAFLD are not rare but represent one significant end of the phenotypic spectrum of NAFLD. We also analyzed the effects of hyperuricmia on NAFLD in non-obese subjects, and observed that hyperuricemia is independently associated with the risk of NAFLD in initially non-obese. As recently as 2012, a study on 11613 participants from United States showed that lean NAFLD was independently associated with younger age, female sex and a decreased likelihood of having IR and hypercholesterolemia[50]. Then, Feng, RN et al. [51] analyzed on 2000 Chinese adults and found that lean NAFLD had comparable triglyceride, cholesterin and low-density lipoprotein cholesterin to overweight-obese individuals, and significantly higher visceral adiposity index than overweight-obese controls. More recently, FeldmanA et al. [52] investigated clinical, genetic, metabolic and lifestyle characteristics in lean Caucasian subjects with NAFLD and found that Lean subjects with evidence of NAFLD have clinically relevant impaired glucose tolerance, low adiponectin concentrations and a distinct metabolite profile with an increased rate of PNPLA3 risk allele carriage. Argo CK and Henry ZH[53] also characterized "lean" NAFLD as a unique phenotype with specific genetic associations deserving of further investigation in the greater scheme of elucidating the pathophysiology of fatty liver.

BMI was confirmed as the most useful predictive factor for NAFLD onset in both sexes in a community-based retrospective longitudinal cohort study [54]. The sensitivity and specificity of body mass index (BMI) as a marker of NAFLD was estimated at 81% and 84%, respectively [55]. Meanwhile, another predictive factor for NAFLD, uric acid, was reportedly responsible for lipid metabolism impairment, including mitochondrial oxidative stress [56], sterol regulatory element-binding protein 1 (SREBP-1) activation induced by endoplasmic reticulum (ER) stress [57], and NLRP3 inflammasome involvement[58]. Thus, there may be an interaction between high SUA levels and obesity in the pathogenesis of NAFLD. Zhang S et al. conducted a cross-sectional study in a Chinese population of 10,069 participants, and reported that obesity and elevated UA levels have significant synergistic effects on the development of NAFLD [59]. Contrasting results for obese and non-obese individuals in our study also confirmed this hypothesis epidemiologically.

According to statistics, 80% of NAFLD occurred in men. We have reason to suspect that the large difference of prevalence of NAFLD may be related to some differences between males and females. As early as 2000, Lonardo, A et al. studied the problem that are there any sex differences in fatty liver and found that glucose metabolism and body fat distribution predicted fatty liver only in women[60]. Suzuki A and Abdelmalek MF summarized known gender differences as well as the proposed mechanisms for gender differences in NAFLD, reviewed that two women-specific issues, menopause and polycystic ovary syndrome, may influence the prevalence and severity of NAFLD[61]. After that, Moon, S. S assessed the association of SUA with NAFLD in pre- and postmenopausal women, respectively, and observed that UA within the normal range showed an association with NAFLD in postmenopausal women, but not in premenopausal women [62]. Two studies [33, 63] focused on Chinese postmenopausal women confirmed that higher SUA levels even within the normal range are positively and independently associated with the presence of NAFLD. Similarly, we found that females with hyperuricemia seem to have higher NAFLD risk than males with hyperuricemia independently of age, BMI, metabolic syndrome components and other clinical variables. This result is consistent with a recent meta-analysis comprising 117,712 subjects [64]. It reported that increased NAFLD risk is markedly associated with hyperuricemia in both men (RR = 1.26, 95% CI1.15–1.37, P<0.001) and women (RR = 2.01, 95% CI1.58–2.56, P<0.001). The differing magnitude of the association may also indicate that SUA participates in the development of NAFLD through different mechanisms in males and females. To the best of our knowledge, no studies have yet suggested what such mechanisms might be. Thus, further study is necessary to be conducted.

One strengthen point of our study is that the chronological order of exposure and outcome make it reasonable to believe that our results may have higher credibility than those drawn from some other study designs. However, the study also had several potential limitations. First, NAFLD was diagnosed based on ultrasonography, which is not sensitive enough to detect mild steatosis. However, ultrasonography is widely used because it is non-invasive, safe, and commonly available. Second, because of the shortcomings of the retrospective study, we could not collect HOMA-IR, waist circumstance and information on dietary habits and lifestyle characteristics which are important factors associated with NAFLD. Thus, we could not assess their impact on the onset of NAFLD. Third, Due to the incomplete collection of anthropometric and biochemical parameters in 2016, we did not assess the impact of potential changes in BMI and metabolic status on NAFLD.

In conclusion, our retrospective cohort study demonstrated that hyperuricemia was independently associated with the development of NAFLD in non-obese subjects but not in obese subjects. The independent effect of hyperuricemia on NAFLD was stronger in females than that in males. Further studies are needed to explore the different mechanisms between obese and non-obese, and the reason why hyperuricemia raises NAFLD risk in females more than in males.

## Supporting information

S1 Checklist STROBE checklist. (DOCX)
