# Supplementary material for: Association between the hyperuricemia and nonalcoholic fatty liver disease risk in a Chinese population: A retrospective cohort study
Source: PLoS One. 2017 May 16;12(5):e0177249. doi: 10.1371/journal.pone.0177249 (PMC5433681; doi:10.1371/journal.pone.0177249)
Supplement: S1 Checklist — (DOCX) [file pone.0177249.s001.docx]

STROBE Statement—checklist of items that should be included in reports of observational studies

|  | Item No. | Recommendation | Page  No. | Relevant text from manuscript |
| --- | --- | --- | --- | --- |
| **Title and abstract** | 1 | (*a*) Indicate the study’s design with a commonly used term in the title or the abstract | 1 | A Retrospective Cohort Study |
|  |  | (*b*) Provide in the abstract an informative and balanced summary of what was done and what was found | 1 | A total of 2383 initially NAFLD-free subjects were followed up for four years, and 15.2% (363/2383) developed NAFLD. lowering uric acid levels in non-obese hyperuricemia subjects is an important step in treating or preventing NAFLD. |
| Introduction | | | |  |
| Background/rationale | 2 | Explain the scientific background and rationale for the investigation being reported | 2 | the mechanism linking SUA and NAFLD had not been fully elucidated. Moreover, to the best of our knowledge, no cohort study specifically examined the relationship between SUA levels and NAFLD in obese subjects.  studies all suggested that high SUA levels are an independent risk factor for NAFLD in non-obese subjects. However, whether this association is causal, a bystander, or a consequence of NAFLD remains under debate. |
| Objectives | 3 | State specific objectives, including any prespecified hypotheses | 2 | we firstly aimed to clarify whether hyperuricemia plays a causal role for NAFLD in obese and non-obese subjects, respectively. Secondly, we investigated whether obesity influences the relationship between NAFLD and SUA. |
| Methods | | | |  |
| Study design | 4 | Present key elements of study design early in the paper |  |  |
| Setting | 5 | Describe the setting, locations, and relevant dates, including periods of recruitment, exposure, follow-up, and data collection | 3 | **Study design and subjects** |
| Participants | 6 | (*a*) *Cohort study*—Give the eligibility criteria, and the sources and methods of selection of participants. Describe methods of follow-up  *Case-control study*—Give the eligibility criteria, and the sources and methods of case ascertainment and control selection. Give the rationale for the choice of cases and controls  *Cross-sectional study*—Give the eligibility criteria, and the sources and methods of selection of participants | 3 | **Study design and subjects** |
|  |  | (*b*)*Cohort study*—For matched studies, give matching criteria and number of exposed and unexposed  *Case-control study*—For matched studies, give matching criteria and the number of controls per case |  |  |
| Variables | 7 | Clearly define all outcomes, exposures, predictors, potential confounders, and effect modifiers. Give diagnostic criteria, if applicable | 3-4 | **Assessment of outcomes and definitions** |
| Data sources/measurement | 8* | For each variable of interest, give sources of data and details of methods of assessment (measurement). Describe comparability of assessment methods if there is more than one group | *3* | ***Baseline examinations*** |
| Bias | 9 | Describe any efforts to address potential sources of bias |  |  |
| Study size | 10 | Explain how the study size was arrived at |  |  |

Continued on next page

| Quantitative variables | 11 | Explain how quantitative variables were handled in the analyses. If applicable, describe which groupings were chosen and why | 4 | Continuous variables are expressed as means±SD or medians and inter-quartile ranges, and were compared using the Student t-test or the Mann-Whitney U test, depending on the normality of the data. |
| --- | --- | --- | --- | --- |
| Statistical methods | 12 | (*a*) Describe all statistical methods, including those used to control for confounding | 4 | Student t-test，Mann-Whitney U test, chi-square test, Cox proportional hazards regression model(univariate model and multivariate model) |
|  |  | (*b*) Describe any methods used to examine subgroups and interactions | 4 | The Cox proportional hazards regression analysis was divided into two steps. First, the analysis was carried out for the total population. Second, the analysis was performed on the two subgroups (non-obese and obese), and a stratified analysis was conducted by gender in each subgroup. |
|  |  | (*c*) Explain how missing data were addressed |  |  |
|  |  | (*d*) *Cohort study*—If applicable, explain how loss to follow-up was addressed  *Case-control study*—If applicable, explain how matching of cases and controls was addressed  *Cross-sectional study*—If applicable, describe analytical methods taking account of sampling strategy |  |  |
|  |  | (*e*) Describe any sensitivity analyses |  |  |
| Results | | | | |
| Participants | 13* | (a) Report numbers of individuals at each stage of study—eg numbers potentially eligible, examined for eligibility, confirmed eligible, included in the study, completing follow-up, and analysed |  |  |
|  |  | (b) Give reasons for non-participation at each stage |  |  |
|  |  | (c) Consider use of a flow diagram |  |  |
| Descriptive data | 14* | (a) Give characteristics of study participants (eg demographic, clinical, social) and information on exposures and potential confounders | 5 | Table 1 |
|  |  | (b) Indicate number of participants with missing data for each variable of interest |  |  |
|  |  | (c) *Cohort study*—Summarise follow-up time (eg, average and total amount) | 5 | The overall four-year cumulative incidence of NAFLD was 15.2% |
| Outcome data | 15* | *Cohort study*—Report numbers of outcome events or summary measures over time | *5* | *Over the four-year study period, 363 subjects (256 male and 107 female) developed NAFLD, a 21.5% and 9.0% cumulative incidence in men and women, respectively. The overall four-year cumulative incidence of NAFLD was 15.2%* |
|  |  | *Case-control study—*Report numbers in each exposure category, or summary measures of exposure |  |  |
|  |  | *Cross-sectional study—*Report numbers of outcome events or summary measures |  |  |
| Main results | 16 | (*a*) Give unadjusted estimates and, if applicable, confounder-adjusted estimates and their precision (eg, 95% confidence interval). Make clear which confounders were adjusted for and why they were included | 6-7 | Table 2-table 4 |
|  |  | (*b*) Report category boundaries when continuous variables were categorized |  |  |
|  |  | (*c*) If relevant, consider translating estimates of relative risk into absolute risk for a meaningful time period |  |  |

Continued on next page

| Other analyses | 17 | Report other analyses done—eg analyses of subgroups and interactions, and sensitivity analyses |  |  |
| --- | --- | --- | --- | --- |
| Discussion | | | | |
| Key results | 18 | Summarise key results with reference to study objectives | 7 | First paragraph of Discussion |
| Limitations | 19 | Discuss limitations of the study, taking into account sources of potential bias or imprecision. Discuss both direction and magnitude of any potential bias | 9 | First paragraph |
| Interpretation | 20 | Give a cautious overall interpretation of results considering objectives, limitations, multiplicity of analyses, results from similar studies, and other relevant evidence | 9 | Second paragraph |
| Generalisability | 21 | Discuss the generalisability (external validity) of the study results |  |  |
| Other information | |  | | |
| Funding | 22 | Give the source of funding and the role of the funders for the present study and, if applicable, for the original study on which the present article is based |  |  |

*Give information separately for cases and controls in case-control studies and, if applicable, for exposed and unexposed groups in cohort and cross-sectional studies.

**Note:** An Explanation and Elaboration article discusses each checklist item and gives methodological background and published examples of transparent reporting. The STROBE checklist is best used in conjunction with this article (freely available on the Web sites of PLoS Medicine at http://www.plosmedicine.org/, Annals of Internal Medicine at http://www.annals.org/, and Epidemiology at http://www.epidem.com/). Information on the STROBE Initiative is available at www.strobe-statement.org.
